# SuSiNE: Genetic fine-mapping with signed functional priors and multi-basin ensembling

**DOI:** 10.64898/2026.07.31.742084

**Published:** 2026-08-12

**Authors:** Michael G. Callahan, Xiang Zhu

## Abstract

Genetic fine-mapping identifies causal variants within trait-associated loci, but linkage disequilibrium (LD) and wide datasets complicate this sparse variable-selection problem. SuSiE is popular for its fast variational inference, posterior inclusion probabilities (PIPs), and credible sets, yet a single fit can fail to resolve LD ambiguity, converge to a poor local optimum, or misrepresent uncertainty over competing configurations. We introduce SuSiNE (Sum of Single Non-central Effects), a SuSiE extension incorporating signed functional annotations through a prior-mean channel, ***µ***_0_ = *c* ***a*** , while preserving effect conjugacy, credible sets, and summary-statistic sufficiency. The resulting single-effect Bayes factor self-gates on agreement between annotation sign and association direction, limiting annotation-noise influence. We show that the common final step of purity filtering can discard informative signal, and tends to hurt performance. We also introduce new effect-level diagnostics for concentration, accuracy, and fitted-basis movement, to provide deeper insights into model behavior. To explore and summarize multiple variational basins, we pair the model with grid-based ensembling and cluster-weight aggregation. In oligogenic simulations with annotations calibrated to AlphaGenome eQTL benchmarks, the ensemble raised pooled AUPRC for recovery of the largest-effect causal variants from a SuSiE-equivalent 0.2474 to 0.3130 (0.0656 delta, 95% paired-bootstrap CI [0.0591, 0.0722]). At 75% precision, recall rose from 11.9% to 19.3% (61.7% relative gain). AUPRC gains were robust across varying annotation quality and alternative sparse and diffuse architectures, while sufficiently strong null annotation-association alignment reversed the gains. In a GTEx Lung summary-statistic case study, SuSiNE placed nontrivial weight on annotation-informed fits at 7 of 20 loci and changed which variants received high PIP. *ARSA* showed the cleanest durable shift, whereas the large *YDJC* shift coincided with reference-LD discrepancy. An internal diagnostic found little evidence of strong annotation confounding in this panel. These analyses use reference rather than in-cohort LD, demonstrating method behavior rather than definitive variant-level discoveries.

**Author summary:**

When a genetic study links part of the genome to a disease or to differences in gene expression, the next question is which variants are responsible. Answering this is hard, because nearby variants are usually inherited together and can look almost interchangeable in the data. We studied a widely used method, SuSiE, by asking where it breaks down. We found that a routine final cleanup step often discards real signal for nothing in return. A single run can also settle on one explanation without exploring alternatives that fit the data just as well. We introduce new checks that make both problems visible. We then developed SuSiNE, which lets the method use directional predictions from AI sequence models or other biological evidence. It runs many times across settings that encourage exploration, then combines the results into one summary. In calibrated simulations, SuSiNE found true causal variants substantially more often than the standard method. On real gene-expression data, it changed which variants look responsible at several locations. These results are limited, but they suggest AI sequence models are already good enough to offer competing explanations at well-studied genome locations, if we use them carefully.

## Full Text Availability

The license terms selected by the author(s) for this preprint version do not permit archiving in PMC. The full text is available from the preprint server.

